# Computer-aided Molecular Design of Compounds Targeting Histone Modifying Enzymes

**DOI:** 10.1016/j.csbj.2015.04.007

**Published:** 2015-05-07

**Authors:** Federico Andreoli, Alberto Del Rio

**Affiliations:** a Department of Experimental, Diagnostic and Specialty Medicine (DIMES), Alma Mater Studiorum, University of Bologna, Via S. Giacomo 14, 40126 Bologna, Italy; b Institute of Organic Synthesis and Photoreactivity, National Research Council, Via P. Gobetti, 101 40129 Bologna, Italy

**Keywords:** Epigenetics, Post-translational modifications, Histone, Computer-aided molecular design, Drug design, Drug discovery

## Abstract

Growing evidences show that epigenetic mechanisms play crucial roles in the genesis and progression of many physiopathological processes. As a result, research in epigenetic grew at a fast pace in the last decade. In particular, the study of histone post-translational modifications encountered an extraordinary progression and many modifications have been characterized and associated to fundamental biological processes and pathological conditions. Histone modifications are the catalytic result of a large set of enzyme families that operate covalent modifications on specific residues at the histone tails. Taken together, these modifications elicit a complex and concerted processing that greatly contribute to the chromatin remodeling and may drive different pathological conditions, especially cancer. For this reason, several epigenetic targets are currently under validation for drug discovery purposes and different academic and industrial programs have been already launched to produce the first pre-clinical and clinical outcomes. In this scenario, computer-aided molecular design techniques are offering important tools, mainly as a consequence of the increasing structural information available for these targets. In this mini-review we will briefly discuss the most common types of known histone modifications and the corresponding operating enzymes by emphasizing the computer-aided molecular design approaches that can be of use to speed-up the efforts to generate new pharmaceutically relevant compounds.

## 1 Introduction

In the last decades, major efforts have been done in genetics though it is evident that unraveling complex biological mechanisms like cancer requires a larger framework of research that recently evolved toward the better understanding of the immune system regulation [1], the rediscovery of metabolism [2] and, importantly, the role of epigenetics [3]. The term epigenetics refers to the mechanisms of temporal and spatial control of gene activity that do not entail modification of the DNA sequence but influence the physiological and pathological development of an organism. The molecular mechanisms by which epigenetic changes occur are complex and cover a wide range of processes [4]. Epigenetic mechanisms can occur at biochemical level in three different ways: i) through histone post-translational modifications (PTMs), which will be the object of this mini-review, as well as the molecular recognition of non-catalytic readers of histones [5], ii) through the DNA methylation, i.e. the methylation of cytosines to 5-methylcytosines, which is the object of recent reviews [6–8], and iii) through regulation of gene expression by non-coding RNA (ncRNA), which is also an emerging topic of research, covered by recent reviews [9–11]. All these processes contribute to define the epigenetic mechanisms by which gene expression is activated or silenced [12–16].

Post-translational modifications of histones occur at the N-terminal tails of the protein chains and consist in covalent modifications that are catalyzed by different classes of enzymes [17,18]. The ensemble of these modifications is commonly referred as to be the *histone code* referring to the idea that all histone PTMs determine the activity state of an underlying gene [19]. One of the hallmarks of the histone code is that it can be positively or negatively correlated with specific transcriptional states or organization of chromatin [20–23]. This is accomplished through a fine regulation of histone PTMs controlled by an enzymatic machinery, which existence and function have been elucidated partly, but with an extraordinary progression in the last years [23–29]. Importantly, further understanding of epigenetic phenomena occurring on histone proteins is critical to shed light on biological processes that are progressively translating into the development of new medical options [29–31]. In this direction, different studies have highlighted how the histone alterations contribute to the onset and growth of a variety of cancers [7,23,24,27,32–41], among other pathologies. Consequently, enzymes operating PTMs on histones are constituting attractive therapeutic targets for the development of new therapies [13,31,42–44]. It should be noted that, while the resulting effects on chromatin collectively depend on the ensemble of histone PTMs, these are operated by precise variations of physicochemical properties that we recently reviewed [17]. For these reasons, large efforts from both academic and industrial settings have been dedicated in the last year to identify and evaluate new biologically active compounds against histone modifying enzymes. Fuelled by the increasing availability of structural information, several endeavors have been initiated and helped by the usage of computer-aided molecular design techniques. Thus, in this mini-review, we aim to highlight the aspects relating histone modifications in the light of the future applications of computational techniques to the research of new probe or lead-like epigenetic modulators.

## 2 Type of Histone Modifications and Their Biological and Clinical Relevance

**Fig. 1:**
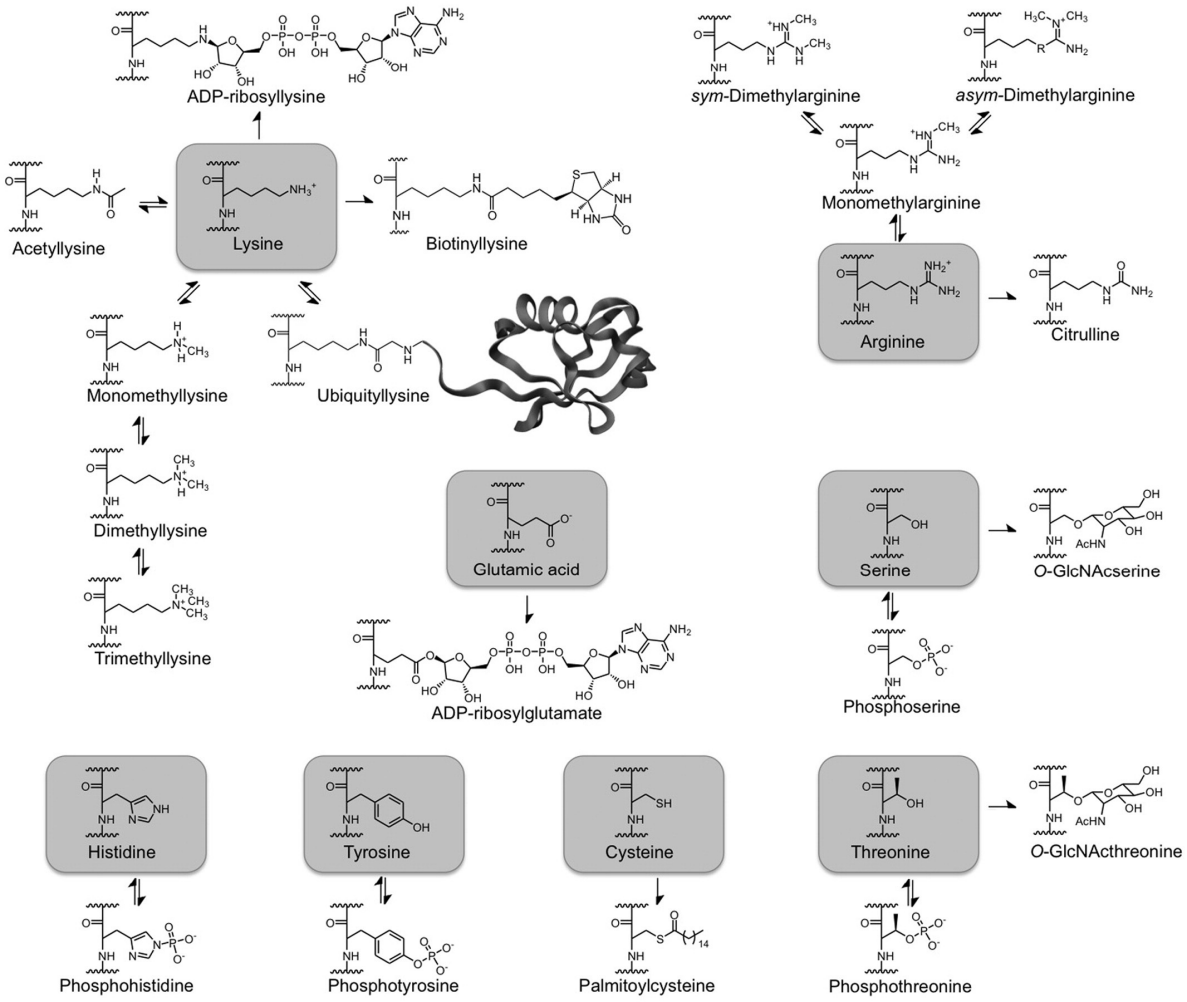
Most common type of post-translational modifications occurring on histone proteins.

To understand the relevance of computational techniques in histone-related epigenetic targets, it is important to highlight that these post-translational modifications are functionalizations/defunctionalizations of specific residues, which are lysine, arginine, serine, threonine, histidine, tyrosine, cysteine and glutamic acid, located at the N-terminal tails of each chain. Fig. 1 summarizes all the most common PTMs that can occur on histones. By far, lysine represents the residues with most chemical versatility, as it is capable to undergo several kinds and grades of modifications. Consequently, histone methyltransferases, demethylases, acetyltransferases and deacetylases have been recently ascribed an important role as new classes of biological targets for drug discovery [18,45–49]. Arginine represents also a residue that is modified by enzymes recognized for drug development, in particular histone methyltransferases. Differently to these previous cases, enzymes that modify histone serines, threonines, histidines, tyrosines, cysteines and glutamic acids have not been exploited yet for the discovery of new modulating compounds. Nevertheless, it is expected that further elucidation of their biological role and protein structure will spur such endeavors. It is worth to note that other kinds of modifications like propionylation, butyrylation, crotonylation, 2-hydroxyisobutyrylation have been reported [50].

Different studies elucidate the impact that PTMs have on chromatin and their relevance in human physiology and pathology [16,18,25,26,31,51–57]. Interestingly, their biological role greatly varies, depending on the kind of modification. Therefore, for instance, the acetylation appears to be the most promiscuous histone modification and is always associated to transcriptional activity. Conversely, histone methylation has a high degree of selectivity toward specific histone residues and can be associated with both repression and transcription [58,59]. In addition PTMs can “cross-talk”, meaning that modifications can occur in a concerted or a subsequent manner [25,60–63].

In the last decade, epigenetic modifications of histones have been mainly studied in the context of cancer, particularly histone deacetylases of classes I, II and IV (HDACs) [64]. Indeed, abnormal activity of the enzymes responsible for deacetylation of histones, modification that alters the chromatin structure repressing transcription, has been shown to be implicated in several diseases [18,65]. Because of these compelling evidences, HDACs have been recognized as consolidated drug target, in particular for breast cancer, colorectal cancer, leukemia, lymphoma, ovarian and prostate cancer. In addition, another class of histone deacetylases named sirtuins (or class III deacetylases), which uses NAD^+^ to catalyze the removal of an acetyl group, also came into the light as new therapeutic targets [66,67]. This family of enzymes, in fact, was found to be involved in relevant physiological and pathological processes, as well as in aging-related disorders, metabolic and inflammatory conditions and processes involving DNA regulation and integrity, including cancer [68–71]. Interestingly, also the correspondent families of enzymes that revert the catalytic activity of histone deacetylation, i.e. acetyltransferases (HAT), have also met a great deal of interest. Two classes of HAT exist and consist of enzymes able to acetylate multiple sites in the histone tails and additional sites on the globular histone core, for the first class, while the second class mostly consists of cytoplasmic enzymes able to acetylate newly synthesized histones prior to their deposition into chromatin. Abnormal regulation of HATs has been linked to leukemia and several studies connecting them to prostate and gastric cancers have been realized [72,73].

The second most studied histone modification is methylation. As anticipated above, protein methyltransferases (PMT) emerged recently as new important targets for cancer therapy, since they were found to be overexpressed or repressed in several types of cancer, precisely in breast cancer, leukemia, myeloma, ovarian, prostate and kidney cancers. Several recently published reviews describe mechanism and biological roles of PMTs [6,41,45,46,74–81]. Indeed, due to their importance in different pathological conditions, several drug discovery programs have been launched in order to design specific compounds able to modulate these targets [46]. Equally, the recent focus in understanding histone methylation led to the characterization of histone demethylases (HDM). The first described protein has been the lysine specific demethylase 1 (LSD1) [82]. Soon after, a new class of proteins having demethylase activity, the JMJC (Jumonji C) domain family, was discovered and characterized [83]. LSD and JMJC demethylases have been reported to be regulators of various cellular processes. Therefore, a special effort, as in the case of PMTs, is currently made, aimed to the discovery of small-molecule inhibitors with therapeutic potential [47,83].

Beside the above classes of enzymes, there is a mounting evidence that also other type of modifications can constitute important paradigm in epigenetics and may underlie new biological target for therapeutic purposes. For instance, some studies highlighted specific roles of ubiquitination [84–87], poly-ADP-ribosylation [88–96] and glycosylation [61,97–101] to the epigenetic code. However, the therapeutic potential of these modifications still needs to be validated for the therapeutic and drug discovery point of view. Equally, other modifications like histone phosphorylation, citrullination (deamination) [102], biotinylation [103,104], tail clipping and proline isomerization [25], are still poorly understood and their role in human pathologies remains largely unclear [25]. In the next paragraph, we aim to describe the state-of-the-art of these modifications, analyzing, from a chemical point of view, their role on the dynamics of histone proteins.

## 3 Chemical Mechanisms of Histone Modifications

The histone code collectively depends on the ensemble of post-translational modifications that is operated at the chromatin level, by single variations of physicochemical properties of the modified residues. Consequently, the microenvironment and the biochemical differences obtained by PTMs depend on the attachment or removal of chemical functional groups, whose enzymes, mechanisms of action and cofactors are overviewed in Table 1. We recently proposed that, from a chemical functionality point of view, histone PTMs can be divided in two main groups [17]. The first group I encompassing PTMs leading to the addition or the removal of monofunctional, generally small, organic substituents and a second group including polyfunctional, and in some case elaborate and large, organic molecules. Both groups are composed of writer or eraser enzymes, i.e. which add or remove from histone residues specific substituents.

Modifications of the first group are acetylation, methylation, phosphorylation, deimination and palmitoylation (acylation). As seen above, acetylation is the most common and studied PTM and consists in the addition of an acetyl group on a lysine residue mediated by two major classes of histone acetyltransferases (HAT), Type-A (which includes GNAT, p300/CBP, and MYST) and Type-B [18,65,72]. This function can be removed by two categories of different catalytic activity enzymes: classes I, II and IV histone deacetylases (HDAC) and class III deacetylases, also known as sirtuins (SIRT) that work with a NAD^+^-dependent mechanism [105].

Methylation is the second most common PTM and lysine, as seen above, is the residue that undergoes the widest number of methylation reactions. Indeed, lysine can be mono-, di-, or tri-methylated by histone methyltransferases (HMT, also known as PKMT). HMTs can also mono- or di-methylate arginines and this class of enzymes is commonly referred to as protein arginine methyltransferases (PRMT) [106]. Methyl groups can be erased only from lysines through the action of histone demethylases (HDM or, in the case of lysines, KDM). Phosphorylation of histone has been observed on serine, threonine [107], tyrosine [107] and histidine [108] sites while phosphatases, which hydrolyze the phosphoric monoesters or monoamides restoring the original residues, represent the erasers of this histone modification. Deimination occurs on arginines and methylarginines through the catalysis of peptidylarginine deiminases (PAD) [109].

Modifications of the second group are ubiquitylation, SUMOylation, biotinylation, glycosylation and ADP-ribosylation. While, in principle, these modifications are catalyzed by writers and eraser enzymes, it is worth to note that decitrullination, depalmitoylation, deADP-ribosylation, debiotinylation and deglycosylation have not been described, yet. Mono- or poly-ADP-ribosylation is catalyzed by ADP-ribosyltransferases (ART), clostridia-toxin-like (ARTC) or diphtheria toxin-like (ARTD), which are most commonly known as PARP, and some sirtuin isoforms (Table 1) [88,110,111]. Biotinylation and glycosylation are still poorly characterized and have been linked to the histone code recently [97,99,112,113]. Ubiquitylation takes place by the action of E1, E2, and E3 enzymes, which catalyze the addition of an ubiquitin (Ub) molecule to the target lysine via an isopeptide bond. The reversal of this action is operated by deubiquitylating enzymes (DUB), so far demonstrated to act only on histones H2A and H2B. SUMO (Small Ubiquitin-like MOdifier) proteins can be added on lysine residues in a similar way.

## 4 Molecular Design Techniques Applied to Histone Modifying Enzymes

Computer-aided molecular techniques are widely used in academia and industrial settings to assist the selection of new compounds that can modulate biological targets. Several examples testify their successful applications in the development of new chemical entities [114–116] and a wide range of disciplines, including chemoinformatics, computational chemistry, structural biology, biophysics, medicinal chemistry, organic chemistry and pharmacology, is at disposal of scientists working in these fields. When applied to the discovery of compounds supposed to become future drugs, these techniques are commonly referred as computer-aided drug design (CADD) techniques. Certainly, among them, virtual screening acquired the greatest popularity due to its ability to screen rapidly and cost-effectively large libraries of chemical compounds [117–119]. CADD techniques can be divided in two categories: ligand-based and structure-based drug design techniques (LBDD and SBDD), even if this classification is becoming nowadays loose, as several techniques offer technical advantages that are proper of both categories. The first category usually takes advantage of the information of known bioactive compounds (ligands), while the second usually exploit three-dimensional structure of the biological target (protein), in order to identify new small-molecule modulators of the protein activity. The growing availability of protein structures resolved by X-ray crystallography or NMR technique has progressively raised the possibility to deploy SBDD. However, ligand-based techniques still constitute useful tools, especially when the structural information of a biological target is missing or when the molecular design effort is not directed toward a target-centric approach. Studies aimed at the modulation of cellular pathways or specific phenotypic traits without a precise knowledge of the mechanism of action are representative of this case.

In the context of epigenetics, the research oriented toward the development of new therapeutically-relevant molecules has flourished in the last years. Several works report rationales, targets, drugs, approaches, compounds, tools and methodologies [32,120]. In addition, different reviews reporting the approaches for the discovery of new *epidrugs* or chemical probes for epigenetic targets have been written recently [18,56,57,121,122]. An intriguing aspect in this field of research is the fast pace aimed to extend the discovery of new compounds to epitargets that are currently not validated and that may offer new perspective for the generation of new therapeutic agents. In this direction, particular aspect of epigenetics, for instance, the modulation of the microRNA biogenesis pathway, have been recognized as new possible way to achieve therapeutic target for human disease, in particular cancer [9,123–125].

**Fig. 2:**
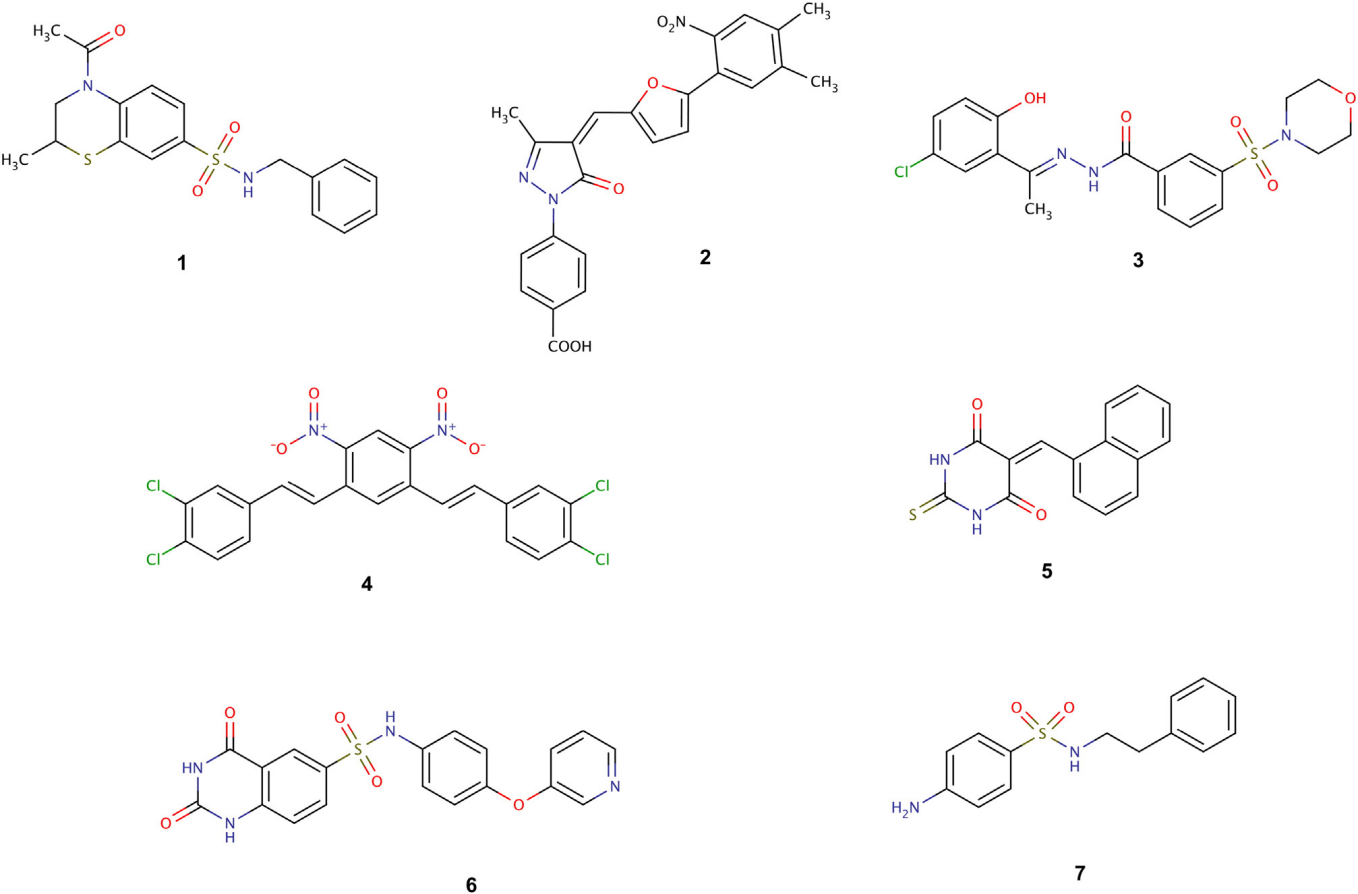
Representative molecular entities able to interfere with histone modifying enzymes. Marvin was used for drawing and displaying chemical structures, Marvin 6.0.0, ChemAxon (http://www.chemaxon.com).

In all this framework of research, computational techniques are playing a progressive role to help the identification of new molecular entities for epigenetic targets. Different techniques have been used to identify new modulating compounds and to explain, mechanism of actions, binding modes and protein dynamics [22,80,126–129]. Most of the research in this direction has been done on classes I, II and IV histone deacetylases. Indeed, a variety of quantitative structure–activity relationship (QSAR) studies and computational works, elucidating retrospectively protein dynamics, binding modes, binding affinities and selectivity among HDAC isoforms, have been published [130–139]. For instance, a recent and prospective work has been done to identify new inhibitors of *Schistosoma mansoni* HDAC8 by means of homology modeling, molecular dynamics and molecular docking techniques [140]. It is interesting to note that most of the prospective works have been applied on other epigenetic targets than HDACs, reflecting the idea that computational techniques are useful tool to help the identification of new biologically active compounds for investigational epigenetic targets. In this direction two molecular docking studies have been applied on HAT p300, resulting on the identification of two classes of new inhibitors: benzothiazines and pyrazolone exomethylene vinyl compounds (**1** and **2**, Fig. 2) [141,142]. Prospective high-throughput docking screening and computational studies with molecular dynamic simulations were performed on LSD1, leading to the identification of nanomolar N′-(1-phenylethylidene)-benzohydrazides (**3**, Fig. 2) and novel classes of short peptide inhibitors [143–146]. LSD1 was also object of retrospective studies based on virtual screening procedures, aiming at elucidating ligand selectivity with a closely related target, the MAO-B [147] and extended molecular dynamic simulations were used to highlight new potential binding regions for drug-like molecules, peptides, protein partners and chromatin [144]. Docking studies were also applied to identify new KDM4B inhibitors, which demonstrated an activity in the low micromolar range of concentration (**4**, Fig. 2), and Sirt1/Sirt2 inhibitors, like thiobarbiturates (**5**, Fig. 2) [148,149]. We also recently applied these techniques to the identification of new and selective inhibitors of Sirt6 (**6**, Fig. 2) [150]. Interestingly, pharmacophore techniques have been used less frequently than docking techniques on histone modifying enzymes. A successful applicative example of the first technique has been published by Sippl and colleagues for the identification of new PRMT1 inhibitors, which were found through a pharmacophore hypothesis built on the basis of the PRMT1–allantodapsone interaction model (**7**, Fig. 2) [151].

Computational chemistry and protein modeling is also actively supporting the basic research on epigenetic targets. A valuable example is the set of tools that are currently used to for detect protein plasticity, dynamics of catalytic sites, analysis of allosteric pockets and interactions with other proteins that may constitute a useful way to study new protein–protein inhibitors (PPI). Some recent reviews discussing these aspects have been written recently on HDACs and other epigenetic players [152–154]. Interestingly, also quantum mechanical methods like QM/MM or DFT have found utility in this field, especially to explore the role metal-containing enzymes and their influence on catalytic activities and the inhibition mechanism by small-molecule modulators [155–157]. Combined computational techniques have been also successfully exploited for the de novo design of inhibitory peptides for histone methyltransferase [158] and chemoinformatic data mining tools like self-organizing maps (SOM) have been exploited for the computational prediction of common non-epigenetic drugs as epigenetic modulators [159]. It is worth to note also that emerging works driven by computational-based techniques are also starting to focus on food and natural components, known to be able to influence the epigenetic code [37,160–164]. Indeed, dietary components like complementary and/or alternative medicines from green tea, genistein from soybean, isothiocyanates from plant foods, curcumin from turmeric, resveratrol from grapes, and sulforaphane from cruciferous vegetables, have been studied for their ability to target the epigenome, especially in different cancer pathologies [18]. Nevertheless, the mechanisms of action of these compounds are still poorly understood and computer-aided techniques are expected to help the comprehension of these mechanisms. In particular, we believe that the availability of compound databases of natural and dietary sources could constitute an effective step toward the identification, development and pharmacological definition of natural and dietary-derived components, affecting epigenetic mechanism, that hold the advantage of pharmaceutical formulation based on natural-occurring scaffold.

## 5 Summary and Outlook

Major research efforts are currently directed toward the discovery of new small-molecules able to modulate epigenetic writers or erases that are involved in chromatin remodeling. Recent successful stories document the possibility to interfere with the epigenetic code with small organic molecules. Indeed, first pre-clinical and clinical results, especially for HDACs, testify that many other *epidrugs* might be effective as combination therapies to control the process of genesis and progression of several forms of cancer, among other pathologies. It is unquestionable that epigenetics framework will play a major role in the near future to develop new therapies against these diseases and molecular design techniques offer the chance to tackle these challenges.

## Figures and Tables

**Table 1:** Mechanisms of histone modifications.

Histone modification	Cofactor	Leaving group	Target residue	Substituents	Operating enzyme	Type of enzyme
Acetylation	Acetyl-CoA	CoA	Lys		HAT	Writer
Deacetylation	Zn^2 +^, Fe^2 +^, Co^2 +^, Mn^2 +^	CH_3_COO^−^	Ac-Lys		HDAC	Eraser
	NAD^+^	*O*AADPr	Ac-Lys		SIRT	Eraser
Palmitoylation	–	–	Cys		–	Writer
Methylation	SAM	SAH	Arg, Lys		PMT	Writer
Demethylation	FAD	HCHO	Me-Lys		LSD	Eraser
			Me_2_-Lys			
	Fe^2 +^, α-KG	HCHO	Me-Lys		PHF8	Eraser
			Me_2_-Lys		JmjD	
			Me_3_-Lys		JHDM	
Citrullination/deimination	Ca^2 +^	NH_3_	Arg		PAD	Writer
			Me-Arg			
Phosphorylation	ATP	ADP	Ser		Kinase	Writer
			Thr			
			Tyr			
			His			
Dephosphorylation	–	–	Phos-Ser		Phosphatase	
			Phos-Thr			
ADP-ribosylation	NAD^+^	NAM	Lys		ADP-ribosyltransferase, SIRT4, SIRT6	Writer
			Glu			
Glycosylation (O-GlcNAcylation)	UDP-GlcNAc	UDP	Ser		OGT	Writer
			Thr			
Biotinylation	–	–	Lys		Biotin-protein ligase	Writer
Ubiquitination	ATP	–	Lys		E1, E2, E3 proteins	Writer
Deubiquitination	–	–	Ub-Lys		Histone H2A deubiquitinase	Eraser
